# Comparison of colour contrast sensitivity in eyes at high risk of neovascular age‐related macular degeneration with and without subsequent choroidal neovascular membrane development

**DOI:** 10.1038/s41433-021-01875-6

**Published:** 2022-01-20

**Authors:** Antonio Calcagni, Olivia Howells, Hannah Bartlett, Alastair K. O. Denniston, Jonathan M. Gibson, Christopher R. Hogg, Timothy D. Matthews, Frank Eperjesi

**Affiliations:** 1 Aston University, School of Life and Health Sciences, Birmingham, UK; 2 University Hospitals Birmingham NHS Foundation Trust, Birmingham, UK; 3 Moorfields Eye Hospital NHS Foundation Trust, London, UK; 4 Academic Unit of Ophthalmology, University of Birmingham, Birmingham, UK; 5 Department of Health Science, Arab American University, Ramallah, Palestine

**Keywords:** Outcomes research, Macular degeneration

## Abstract

**Background:**

Neovascular age‐related macular degeneration (nAMD) is a leading cause of blind registrations in the elderly. Unfortunately, it is difficult to detect the early stage of the disease, when treatment is more likely to be successful. Subjects with very early disease are likely to have abnormal macular function, even in the pre‐symptomatic stage. In this study, colour vision was evaluated to establish if subjects at high risk of developing nAMD can be identified, thus allowing earlier diagnosis and possible treatment.

**Methods:**

Colour contrast sensitivity (CCS) was evaluated over time in the fellow unaffected eye of subjects with unilateral nAMD. Participants were divided into Group 1 (182 participants) or Group 2 (15 participants) according to whether nAMD did not or did develop in the study period respectively and the two groups were compared.

**Results:**

CCS was increased (i.e. worse colour vision) compared with the age-matched reference range in a high proportion of fellow eyes in both Groups 1 and 2. Global mean CCS values did not show statistically significant differences between the two groups. However, there was a statistically significant difference between mean Group 1 CCS values and the last CCS value prior to nAMD diagnosis from Group 2 subjects.

**Conclusion:**

This study shows that in patients with unilateral nAMD, colour vision is frequently abnormal in the fellow unaffected eye. Abnormal CCS does not predict the development of nAMD within the 12 month period of the study and therefore it is not a viable screening tool for this pathology.

## Introduction

Vision loss significantly impacts on quality of life, to a much greater extent than appreciated by many health professionals [1–3]. Neovascular age‐related macular degeneration (nAMD) is one of the leading causes of visual impairment certifications in the UK [4], with prevalence estimated at 2.5% in the over 65s and 6.3% in those aged 80 or more [5]. It is therefore likely to become a much more significant issue as the population ages [5] unless steps are taken to find ways of limiting vision loss.

The advent of anti-vascular endothelial growth factor (anti‐VEGF) therapy in nAMD [6, 7] has dramatically improved the prognosis of patients with nAMD: vision can frequently be maintained or even improved, particularly if early detection and treatment occur [6–9]. However, it is currently difficult to detect and monitor nAMD in its early stages, and most individuals typically present to the ophthalmologist only once a certain degree of irreversible damage has occurred.

The hallmark of nAMD is a choroidal neovascular membrane (CNV) [4, 10, 11], although changes occur locally before a CNV develops, as follows:

1. Inflammatory mediators are released in the affected area and changes in metabolism develop [12–15].
2. The retina affected by the insult gradually loses its function [10–12, 14–18].

The contralateral eye of patients with unilateral nAMD has a 12–15% yearly risk of developing a CNV [19]. It has yet to be established at which point of CNV development the surrounding retina has detectable functional abnormalities. However, a wealth of literature [10–12, 14–18, 20] suggests it starts when the patient is still asymptomatic and before currently available non‐invasive imaging techniques are able to detect any abnormality. Many studies [10, 12, 17, 18, 21–23] indicate that the above-mentioned changes in metabolism determine abnormal function in the nearby photoreceptors/inner retina, even in the earliest stages of the disease process; in particular, some cone populations appear more affected than others [18, 21, 24].

Treatment initiated before the development of structural damage has occurred would most likely allow good levels of vision to be maintained, avoiding the significant personal suffering and socio‐economic burden associated with sight loss from nAMD. In current clinical practice, however, treatment is commenced only once relatively significant structural changes in the retina are obvious on clinical evaluation, fundus photography or optical coherence tomography (OCT).

In the present study, the investigators evaluated whether colour contrast sensitivity (CCS) as measured with the ChromaTest (CH Electronics—UK) [25, 26] is effective in identifying the early, pre‐symptomatic stage of nAMD.

## Materials and methods

Macular and paramacular retinal function was evaluated over time by measuring CCS in the fellow unaffected eye of individuals with unilateral nAMD and compared with the clinical evolution (based on OCT and fundoscopy) in that eye, to establish whether CCS can effectively be used in the clinical setting to detect nAMD at a pre‐clinical stage.

### Study design and methods for the research

In this prospective longitudinal study, all subjects within the cohort of patients attending three different UK hospitals receiving treatment with intravitreal anti‐VEGF were screened. In excess of 3000 potential participants were screened across the three sites and over 500 who met the inclusion criteria were approached (telephonically or directly in the clinics).

### Inclusion and exclusion criteria

Subjects over 50 years of age with unilateral (treated or inactive) nAMD were eligible to take part in the study. Individuals with significant media opacities (defined as the impossibility to adequately assess the retina with a 90 dioptre lens on slit lamp biomicroscopy), visual acuity worse than 0.3 LogMAR on the first visit, bilateral AMD, other retinal pathology that could affect CCS, past ophthalmic history of inherited colour vision deficiency, high refractive error (≥7 dioptres spherical equivalent), and/or not fluent in the English language or unable to give informed consent were not eligible to take part in this study.

Informed written consent was obtained from all eligible participants who accepted to take part, all applicable institutional and governmental regulations were followed (Ethics Approval: West Midlands NRES Committee; study 14/WM/0035), and the study adhered to the tenets of the Declaration of Helsinki. Each participant was assessed at least once. After the first assessment, each successive assessment was carried out at the first suitable clinical visit that occurred more than 55 days from the previous assessment, in order to allow appropriate monitoring of clinical evolution.

### Assessments

**Fig. 1:**
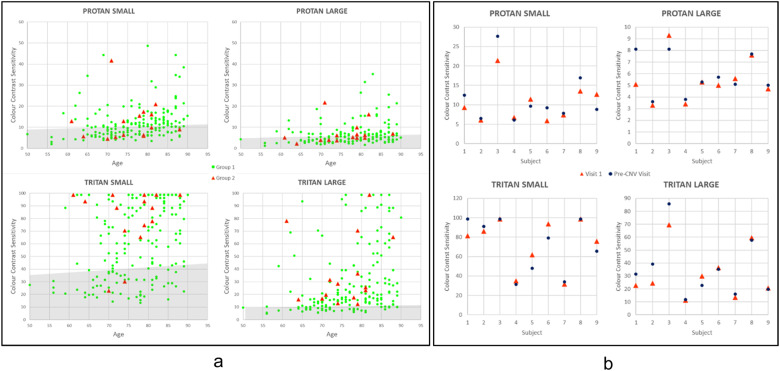
Colour Contrast Sensitivity (CCS) values. **a** CCS values versus age at visit 1 (shaded area shows the CCS mean + 2 SD of individuals with no evidence of retinal pathology in either eye). **b** Comparison between Group 2 early pre-diagnosis mean CCS values (defined as values measured more than three months before nAMD diagnosis was made) and late pre-diagnosis CCS values (measured less than three months before nAMD diagnosis was made).

At each visit, monocular visual acuity was assessed and CCS measured only in the eye not affected by nAMD. If at the first assessment, unexpectedly high CCS values were recorded or if, at subsequent assessments, CCS values were more than 10% higher (i.e., worse colour vision) compared with the previous visit, at least one repeat of the test was performed within the session to ensure reliability of results; furthermore, if more than one assessment of CCS was performed in the same session for the above-mentioned reasons and the intra-sessional variability was excessive, results from that visit were not included in the final analyses. As this study assessed whether deterioration of CCS could be used as a marker of nAMD development, if at the first visit CCS was not measurable in any of the four sub-tests (see below), the individual was excluded from the cohort of participants as it would not be possible to evaluate if CCS changed over time. Four separate sub-tests of CCS assessment were therefore performed, as previously described [25, 26]: protan small, tritan small, protan large and tritan large. Protan tests assess the red/green colour axis, tritan tests assess the blue/yellow colour axis [25]. The ChromaTest has been extensively described previously [18, 27–34]. In brief, all ChromaTest assessments present, in the centre of a calibrated monitor, an isoluminant letter on a neutral background of 20 cd/m^2^ (Fig. S1; Supplementary Material). Random dynamic luminance noise masks any luminance clues that may help recognise the optotype (CCS is not affected by this luminance masking [35]). If the patient correctly identifies the optotype, the colour contrast between letter and background is halved; if the answer is incorrect, the contrast is doubled. Incorrect responses prolong the test, but do not influence the final threshold. The protan and tritan colour confusion lines along which the colours are modulated form the major and minor axes of a MacAdam ellipse centred on white [36]. Small letter tests evaluate the central macular area by subtending 1.5 degrees of visual angle; large letter tests assess paramacular areas by subtending 4 degrees of visual angle. Colour contrast was defined as 0% when the letter had the same hue as the background and 98.7% (due to hardware limitation in obtaining an actual 100% contrast) when the difference in colour between the letter and the background was at its maximum achievable by the monitor; CCS was determined as the minimum contrast required to identify the letter correctly. A higher CCS value therefore indicates poorer colour discrimination. Values obtained from participants were compared with an age-corrected normative database from subjects with no known ocular pathology and values more than 2 standard deviations higher than the age-matched mean [25] were considered significant (Fig. 1a).

After CCS was measured, the assessors established, based on the clinical data available, whether there was any evidence of development of nAMD in the previously unaffected eye, for example intraretinal/subretinal haemorrhage, OCT evidence of intraretinal/subretinal fluid, onset of new symptoms like metamorphopsia/deterioration of vision or retinal thickening in the presence of a lesion compatible with a CNV, then confirmed on fundus fluorescein angiography.

### Data analysis

To establish if CCS is a suitable screening tool for the development of nAMD, participants included in the analysis were divided into two groups: Group 1 did not develop nAMD in the tested eye in the study period (i.e. at least 3 months from the final CCS assessment), whereas Group 2 included all participants who developed nAMD within 3 months of their most recent CCS evaluation (Fig. 1a).

Microsoft Excel and IBM SPSS were used for data analysis. Simple calculations were used to derive mean individual standard deviations and coefficients of variation (CV).

Repeatability was determined using Bland–Altman analysis and only increases or decreases greater than the coefficient of repeatability (CR) were classed as clinically relevant.

The data sets involved did not meet the assumptions of normality and therefore non-parametric statistical tests were used to determine differences or relationships between groups. When comparing two groups, a Mann–Whitney *U* test was employed. When comparing more than two groups, a Kruskal––Wallis test was used. When examining relationships between groups, Spearman’s rho correlations were employed.

For all analyses, a *p* value of less than 0.05 was considered statistically significant and the two-tailed significance level was used.

Statistical analysis was conducted on 197 participants and the total number of assessments over the study period was 884. A summary of the participants’ attendances is given in Table S1 Supplementary Material. The mean inter-assessment interval was 110 days (range: 56–329 days; standard deviation: 54 days).

Group 1 (non-nAMD Group) included 182 participants, whereas Group 2 (nAMD Group) included 15 participants. Excluding the participants with only one visit, the average follow-up time from the first to the last visit within the study was 393 days (range: 56–665 days; standard deviation: 162 days).

## Results

CCS was increased (i.e., colour vision was worse) compared with the age-matched reference range in a high proportion of fellow eyes in all four test conditions (Fig. 1a).

Group 1 CCS values were analysed and subsequently compared with Group 2 CCS values (Fig. 1a; Tables 1 and 2). Average CCS values were calculated across all visits. Excessively variable data within a visit were excluded (*N* = 4), as was one set of data from a participant diagnosed with diabetes mellitus within 6 months of the visit.

**Table 1:** **a**: Summary of Group 1 and Group 2 age, visual acuity and CCS values. **b:** Comparison of average CCS, age and visual acuity between Groups 1 and 2 .

(a)												
	Age		LogMAR VA		Protan small		Tritan small		Protan Large		Tritan large	
Group	1	2	1	2	1	2	1	2	1	2	1	2
*n*	182	15	182	15	178	15	182	15	178	15	181	15
Mean	77.7	76.1	0.13	0.13	12.4	13.0	61.6	76.5	6.5	7.1	28.1	36.3
SD	8.2	6.9	0.10	0.10	8.4	9.5	29.2	24.6	5.2	5.1	24.8	25.5
Median	79	78	0.14	0.16	10.5	10.1	62.6	86.4	4.9	5.4	18.6	27.6
Minimum	51	62	−0.10	−0.01	2.5	4.7	13.8	32.1	1.4	3.2	4.8	11.5
Maximum	90	88	0.30	0.30	64.1	41.1	98.7	98.7	36.6	21.6	98.7	98.7

(b)						
	Protan small	Tritan small	Protan large	Tritan large	Age	LogMAR VA
Group 1	12.4 ± 8.4% (*n* = 178)	61.6 ± 29.2% (*n* = 182)	6.5 ± 5.2% (*n* = 178)	28.1 ± 24.8% (*n* = 181)	77.7 ± 8.2 (*n* = 182)	0.13 ± 0.10 (*n* = 182)
Group 2	13.0 ± 9.5% (*n* = 15)	76.5 ± 24.6% (*n* = 15)	7.1 ± 5.1% (*n* = 15)	36.3 ± 25.5% (*n* = 15)	76.1 ± 6.9 (*n* = 15)	0.13 ± 0.10 (*n* = 15)
*U*	1328	956	1207	951	1157	1355
*p* value	0.975	0.054	0.539	0.054	0.327	0.964

**Table 2:** Comparison between Group 1 average CCS and Group 2 CCS just before nAMD diagnosis.

	Protan Small	Tritan Small	Protan Large	Tritan Large
Group 1	12.4 ± 8.4% (*n* = 178)	61.6 ± 29.2% (*n* = 182)	6.5 ± 5.2% (*n* = 178)	28.1 ± 24.8% (*n* = 181)
Group 2	13.8 ± 9.7% (*n* = 15)	76.2 ± 24.9% (*n* = 15)	7.2 ± 5.1% (*n* = 15)	38.2 ± 27.4% (*n* = 15)
*U*	1274	928	1163	921
*p* value	0.771	0.039	0.409	0.039

### CCS as a screening tool

#### CCS comparisons between Group 1 and Group 2

There were no statistically significant differences in age, visual acuity and mean CCS (all visits) between Groups 1 and 2 (Table 1). Comparison of mean CCS values in Group 1 with the measurements prior to diagnosis in Group 2 (last visit only), revealed significant differences (*p* < 0.05) for both small and large tritan stimuli, but not for protan stimuli (Table 2).

#### Group 2 analysis—“early” pre-diagnosis CCS versus “late” pre-diagnosis CCS

To establish if CCS can be used as a screening tool for the detection of early nAMD, Group 2 CCS values obtained at least 3 months prior to diagnosis were compared with values obtained within 3 months of diagnosis. The low number of participants in this subanalysis (nine fellow eyes) precluded statistical analysis, but there were no consistent differences between the two subgroups (Fig. 1b).

### Intersession repeatability and variability

**Fig. 2:**
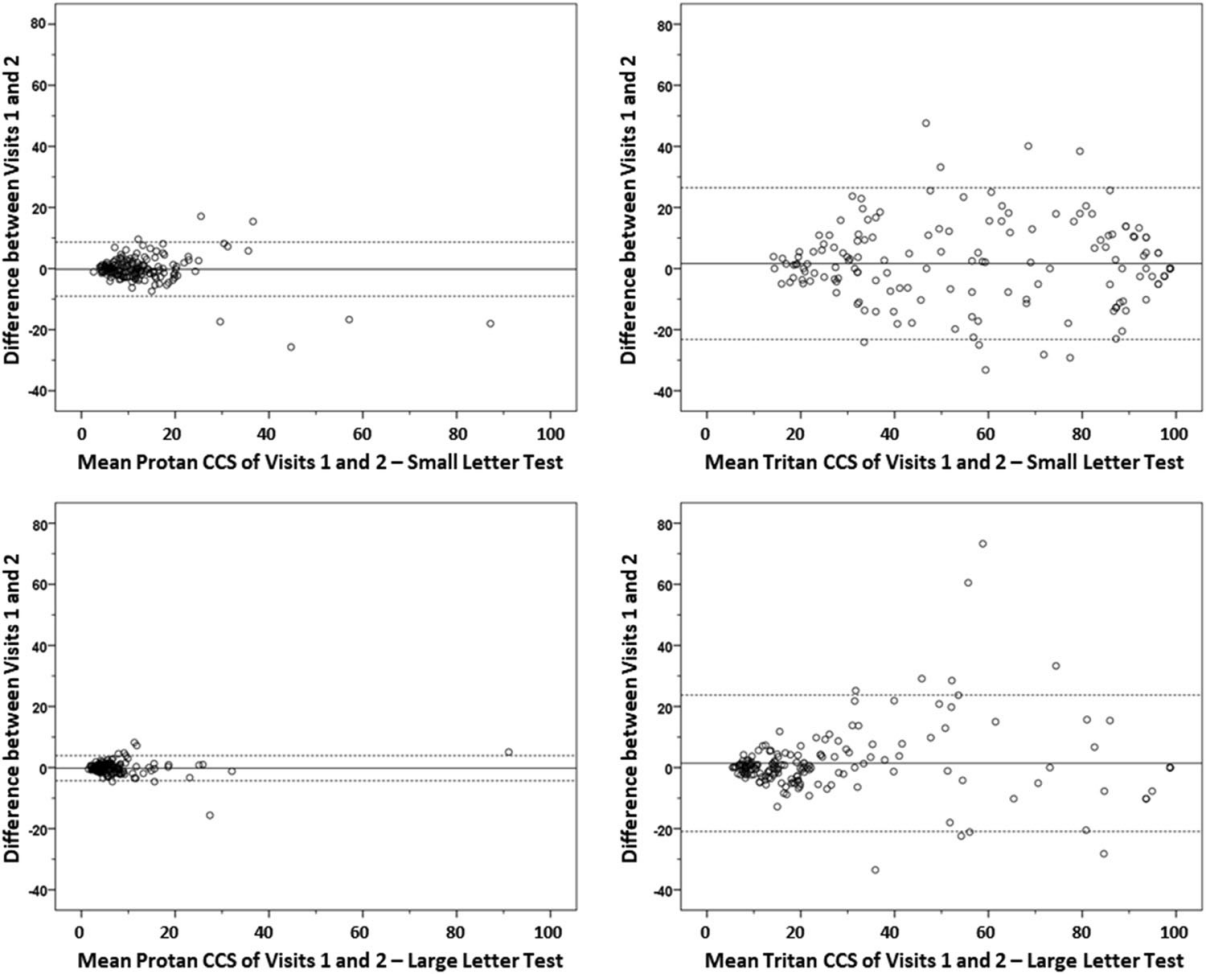
Bland–Altman plots representing the difference in CCS readings between visits one and two, compared with the mean of both visits (*n* = 171 for protan and *n* = 173 for tritan). The solid line represents the mean difference, and the dashed lines represent the 95% confidence limits.

The CR was calculated between visit 1 and visit 2 from all subjects in Group 1 with two or more attendances (protan = 171; tritan = 173; Q64 and S55 protan results, included in the original analysis, were excluded due to outlier-style variability). The CR was 8.8% for the protan small letter test; 24.9% for the tritan small letter test; 4.1% for the protan large letter test; and 22.3% for the tritan large letter test. The CR for all four tests between visit 2 and visit 3 was similar to the CR between visit 1 and visit 2. Figure 2 shows Bland–Altman plots representing the difference in CCS readings between visits 1 and 2, compared with the mean of both visits.

Analysing data from Group 2, the variability over time does not appear to be significantly different from the CR in Group 1. Furthermore, CCS variability from Group 2 does not appear to be consistently or regularly higher or lower than Group 1: this holds true when assessing each individual visit from Group 2 subjects and mean Group 2 CCS values, averaged across all visits (results not shown).

Table 3 shows the means of the individual standard deviations and the individual CV across all Group 1 visits. Mean individual standard deviations were calculated using the standard deviation of each individual’s CCS values, and then the mean of all these. Mean individual CV were calculated from the aforementioned individual standard deviations, divided by the mean of an individual’s CCS values (and multiplied by 100 for the percentage); the mean of all these was then calculated.

**Table 3:** Individual standard deviations and the individual coefficients of variation (CV) across all visits in Group 1 (see text for further details).

	Mean SD (±SD) between visits (min 2 visits, max 9)	Mean CV (±SD) between visits (min 2 visits, max 9) (%)
Protan small	2.3 ± 2.3	18.4 ± 9.1
Tritan small	7.7 ± 6.1	16.2 ± 12.8
Protan large	1.2 ± 1.2	17.9 ± 10.2
Tritan large	5.2 ± 5.8	20.0 ± 13.7

## Discussion

In the present study, the investigators evaluated whether CCS is an effective screening tool to identify the early, pre‐symptomatic stage of nAMD. Participants were divided into Group 1 or Group 2 according to whether nAMD did not or did develop in the study period respectively and the two groups were compared. There was no overall significant CCS difference between Group 1 and Group 2 in the study period, nor does the CCS show any repeatable trend between early and late pre-diagnosis values in Group 2, and therefore CCS is unlikely to be a suitable screening tool for the development of nAMD.

The tritan CCS in the non-affected eye of Group 1 (Fig. 1a) is frequently significantly higher than expected when compared with the normative database [25] of age-matched subjects with no evidence of macular pathology (i.e., both eyes pathology-free); indeed, in many cases, especially for the small letter test, no tritan colour vision could be detected at all. This is in agreement with other studies [21] and although the data from this study suggests this is not relevant for the development of subsequent pathology in the short term (<12 months), the evidence of an abnormal blue/yellow colour axis in the absence of visible maculopathy is likely to be of clinical significance, especially given the associated disease in the fellow eye. CCS colour vision testing may therefore not be of prognostic value within the 12 months of the study but may still have a role in the evaluation of patients at high risk of macular pathology; a further review of data at 5 years from the end of the current study is planned.

It is clear that due to the significant variability of CCS in the unaffected eye of subjects in Group 1, there is no specific value above which the subject is likely to develop nAMD in the short term. It is tempting to speculate that variability in CCS between visits is more relevant, as it suggests the macula in question is unable to compensate for environmental factors such as exposure to light just prior to CCS measurement (a “healthy” retina easily compensates for environmental changes and therefore produces similar results over time).

The statistically significant differences in CCS levels for tritan small and tritan large letter tests (*p* < 0.05) when comparing mean CCS values from Group 1 with CCS values from Group 2 at the last visit prior to nAMD diagnosis (Table 2) and the variability when comparing Group 2 early pre-diagnosis mean CCS values with late pre-diagnosis CCS values (Fig. 1b), need to be interpreted with caution, as too few data over too short an interval are available to establish prognostic value beyond 12 months and further work is required to establish if these findings are of any clinical significance.

### Repeatability and variability of CCS

Protan tests are more repeatable than tritan tests, but this may be due to the high tritan thresholds in the fellow unaffected eye of many subjects with unilateral nAMD and the manner in which the ChromaTest calculates CCS when the contrast needed to see the optotype is high. The variability of CCS throughout the study was similar to that measured in other studies [18, 20, 21, 24]. Some subjects had higher variability than others from visit to visit, but this had no prognostic implications within the study period.

### Future plans for taking the research forward

Further work is needed to establish if patients with a relatively variable CCS have a higher incidence of progression to macular pathology beyond the short duration of this study compared with those who have a reasonably stable CCS between sessions, as it may allow to differentiate which patients need closer monitoring of the fellow unaffected eye and therefore should not be discharged from the clinic.

## Conclusion

This study shows that in patients with unilateral nAMD, there is a high incidence of abnormal colour vision in the fellow unaffected eye. Comparison of tritan and protan CCS suggests the S-cone pathway is most vulnerable to early dysfunction in fellow eyes. Abnormal colour vision does not predict the development of nAMD within the 12 month period of the study, but longer term prognostic value has yet to be established.

## Summary

### What was known before

- Changes in retinal metabolism secondary to a choroidal neovascular membrane determine abnormal function in the nearby photoreceptors/inner retina.
- Retinal function is likely to be affected in pre-symptomatic neovascular age-related macular degeneration.
- Retinal function can be measured with colour contrast sensitivity.

### What this study adds

- Colour contrast sensitivity in the fellow unaffected eye of many individuals with unilateral nAMD is high (i.e., colour vision is poor) and many patients have fluctuating retinal function over time.
- Colour contrast sensitivity is not a tool suitable for screening for choroidal neovascular membranes but may be useful in informing which patients with unilateral nAMD need closer follow up for the fellow unaffected eye.
- Colour contrast sensitivity may be of use for flagging individuals at risk of macular/retinal disease.

## Supplementary information

Supplementary Material Legends

Table S1

Figure S1
