# Supplementary material for: Comparison of colour contrast sensitivity in eyes at high risk of neovascular age‐related macular degeneration with and without subsequent choroidal neovascular membrane development
Source: Eye (Lond). 2022 Jan 20;37(2):297–302. doi: 10.1038/s41433-021-01875-6 (PMC9873808; doi:10.1038/s41433-021-01875-6)
Supplement: Supplementary file 1 — Supplementary Material Legends [file 41433_2021_1875_MOESM1_ESM.docx]

**Supplementary Material Legends**

**Table S1:**

Summary of participants’ attendances during the study period

**Figure S1:**

Examples of ChromaTest Optotypes for the four subtests

(a: protan large high contrast; b: protan large intermediate contrast; c: tritan large high contrast; d: tritan large intermediate contrast)
