# Supplementary material for: Comparison of colour contrast sensitivity in eyes at high risk of neovascular age‐related macular degeneration with and without subsequent choroidal neovascular membrane development
Source: Eye (Lond). 2022 Jan 20;37(2):297–302. doi: 10.1038/s41433-021-01875-6 (PMC9873808; doi:10.1038/s41433-021-01875-6)
Supplement: Supplementary file 2 — Table S1 [file 41433_2021_1875_MOESM2_ESM.docx]

| TOTAL PARTICIPANTS | **197** |
| --- | --- |
| 1 VISIT | 12 |
| 2 VISITS | 20 |
| 3 VISITS | 32 |
| 4 VISITS | 33 |
| 5 VISITS | 43 |
| 6 VISITS | 30 |
| 7 VISITS | 20 |
| 8 VISITS | 6 |
| 9 VISITS | 1 |

Table S1
