# Supplementary figures and images for: Comparison of colour contrast sensitivity in eyes at high risk of neovascular age‐related macular degeneration with and without subsequent choroidal neovascular membrane development
Source: Eye (Lond). 2022 Jan 20;37(2):297–302. doi: 10.1038/s41433-021-01875-6 (PMC9873808; doi:10.1038/s41433-021-01875-6)

**
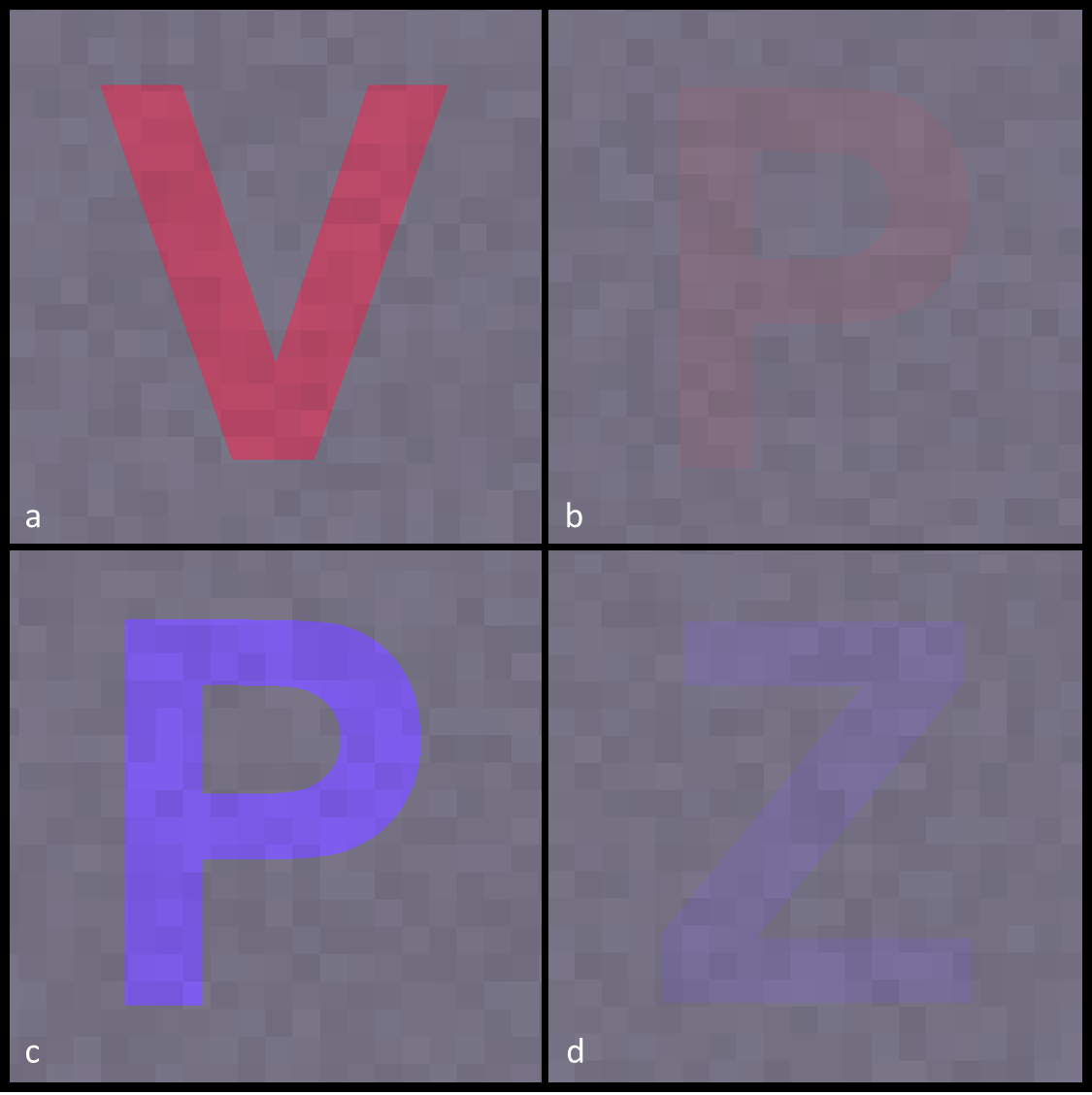
**

Figure S1

Supplement: Supplementary file 3 — Figure S1 [file 41433_2021_1875_MOESM3_ESM.docx]
